# Supplementary material for: Partner independent fusion gene detection by multiplexed CRISPR-Cas9 enrichment and long read nanopore sequencing
Source: Nat Commun. 2020 Jun 5;11:2861. doi: 10.1038/s41467-020-16641-7 (PMC7275081; doi:10.1038/s41467-020-16641-7)
Supplement: Supplementary file 2 — Supplementary Information [file 41467_2020_16641_MOESM2_ESM.pdf]

Supplementary Figures to:

Partner independent fusion gene detection by multiplexed CRISPR/Cas9 enrichment and long-read Nanopore sequencing

Christina Stangl[1,2,3], Sam de Blank[1], Ivo Renkens[1], Liset Westera[4,5], Tamara Verbeek[1], Jose Espejo Valle-Inclan[1,3], Rocio Chamorro González[6,7], Anton G. Henssen[6,7,8,9], Markus J. van Roosmalen[3,4], Ronald W. Stam[4], Emile E. Voest[2,3], Wigard P. Kloosterman[1,10,11], Gijs van Haaften[1+] and Glen Monroe[1+$]

Affiliations

[1] Department of Genetics, Center for Molecular Medicine, University Medical Center Utrecht and Utrecht University, Utrecht, The Netherlands

[2]Division of Molecular Oncology, Netherlands Cancer Institute, Plesmanlaan, Amsterdam, The Netherlands

[3] Oncode Institute

[4] Princess Máxima Center for Pediatric Oncology, The Netherlands

[5] Dutch Childhood Oncology Group (DCOG), The Netherlands

[6] Department of Pediatric Oncology/Hematology, Charité-Universitätsmedizin Berlin, Germany

[7] Experimental and Clinical Research Center (ECRC) of the MDC and Charité Berlin, Berlin, Germany

[8] German Cancer Consortium (DKTK), Partner Site Berlin, and German Cancer Research Center (DKFZ), Heidelberg, Germany

[9] Berlin Institute of Health, Berlin, Germany

[10] Cyclomics, Utrecht, The Netherlands

[11] Frame Cancer Therapeutics, Amsterdam, The Netherlands

+ These authors jointly supervised this work

$ Corresponding Author

Corresponding Author email address: g.monroe@umcutrecht.nl

Keywords: Fusion Genes, Targeted Enrichment, Nanopore Sequencing

A

3′ unknown sequence

5′ ATG............AGGATGCTCCATGCTAGCTAGCGGCTAGGCTAGGGACTAATC 3′

3′ TAC............TCCTACGAGGTACGATCGATCGCCGATCCGATCCCTGATTAG 5′

crRNA sequence: 5′TGCTAGCTAGCGGCTAGGCT3′

Read direction: Downstream

B

5′ unknown sequence

5′ ATG............AGGATGCTCCATGCTAGCTAGCGGCTAGGCTAGGGACTAATC 3′

3′ TAC............TCCTACGAGGTACGATCGATCGCCGATCCGATCCCTGATTAG 5′

crRNA sequence: 5′AGCCTAGCCGCTAGCTAGCA3′

Read direction: Upstream

Supplementary Figure 1:

Examples of crRNA design for directional sequencing are shown. **(A)** crRNAs (grey) are designed containing the sequence of the forward strand of the target gene 20 bp prior to a PAM-sequence (NGG, red underlined) if the unknown gene partner is downstream (3’) of the known target sequence. **(B)** crRNAs (grey) are designed containing the sequence of the reverse strand of the target gene 20 bp prior to a PAM-sequence (NGG, red underlined) if the unknown gene partner is upstream (5’) of the known target sequence.

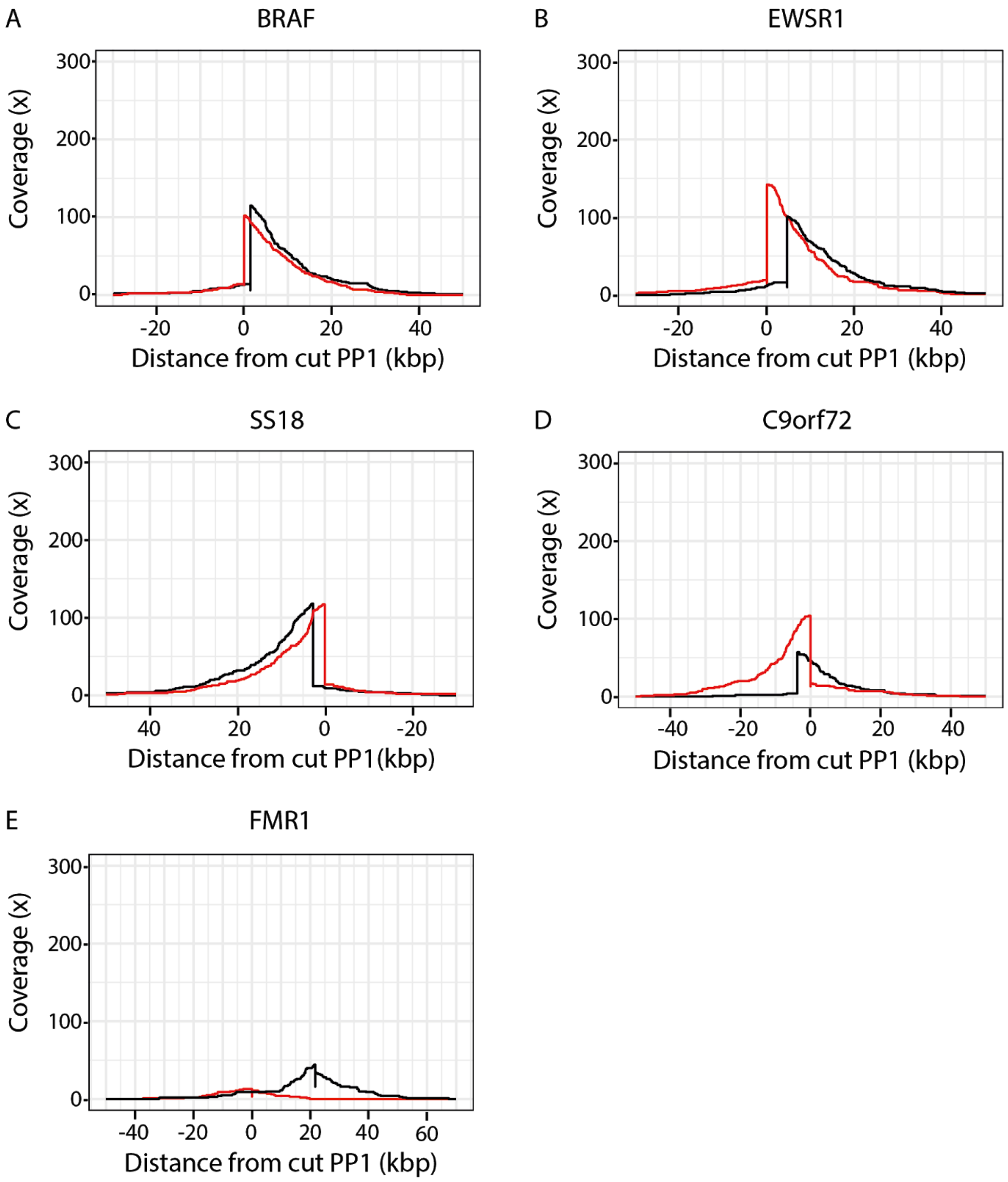

<u>Supplementary Figure 2:</u>

(A-E) Coverage plots showing deconvoluted on-target coverage across multiple genomic loci for two different cut positions (red = PP1 and black = PP2).

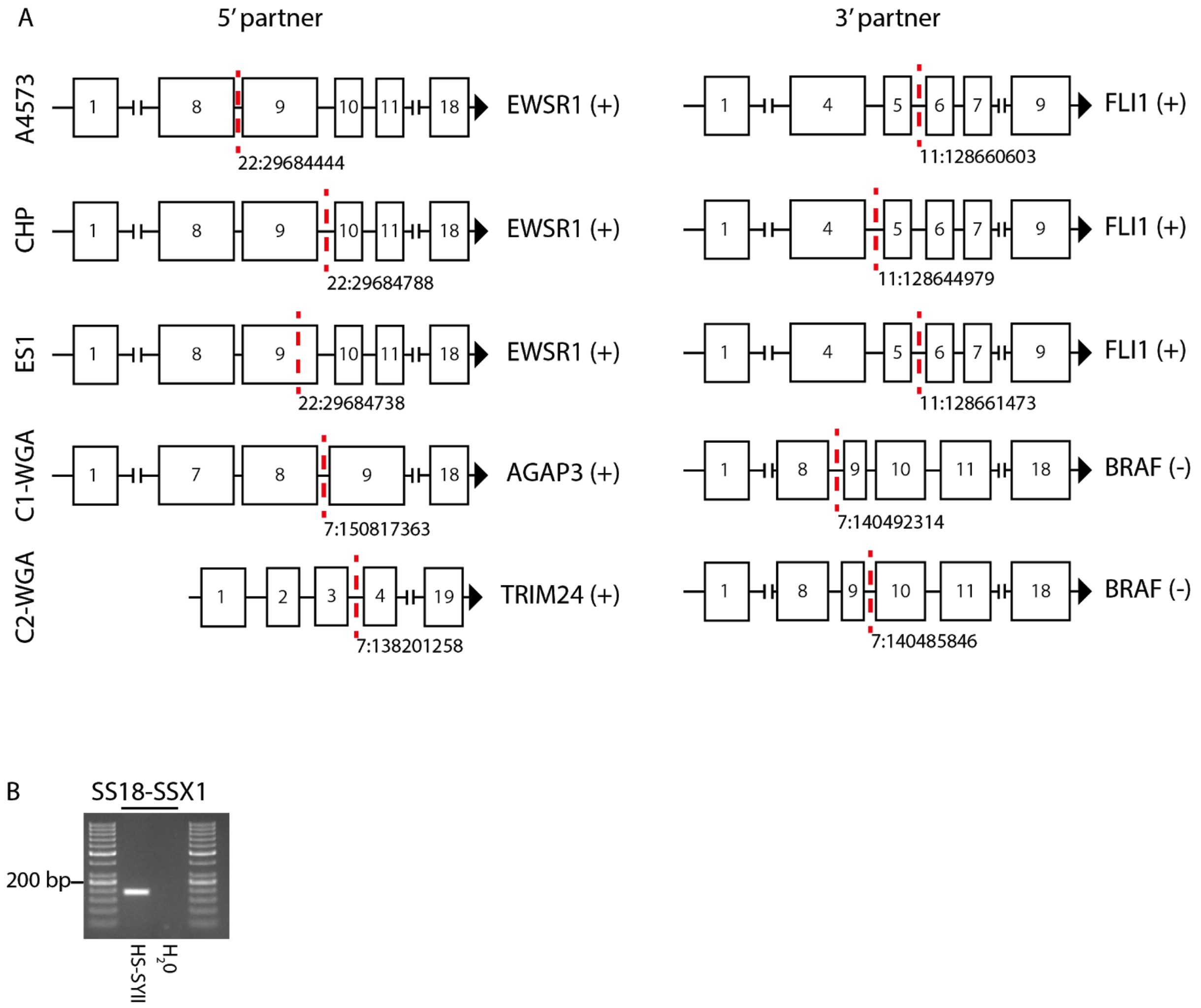

Supplementary Figure 3:

(**A**) Fusion gene configurations for the identified *EWSR1-FLI1*, *AGAP3-BRAF* and *TRIM24-BRAF* fusion genes. (+) or (-) indicate genomic location on the forward or reverse strand of the gene, respectively. Dashed lines (red) indicate break-position for each sample. (**B**) Breakpoint PCR (n=1) for the *SS18-SSX1* fusion gene in the HS-SYII cell line.

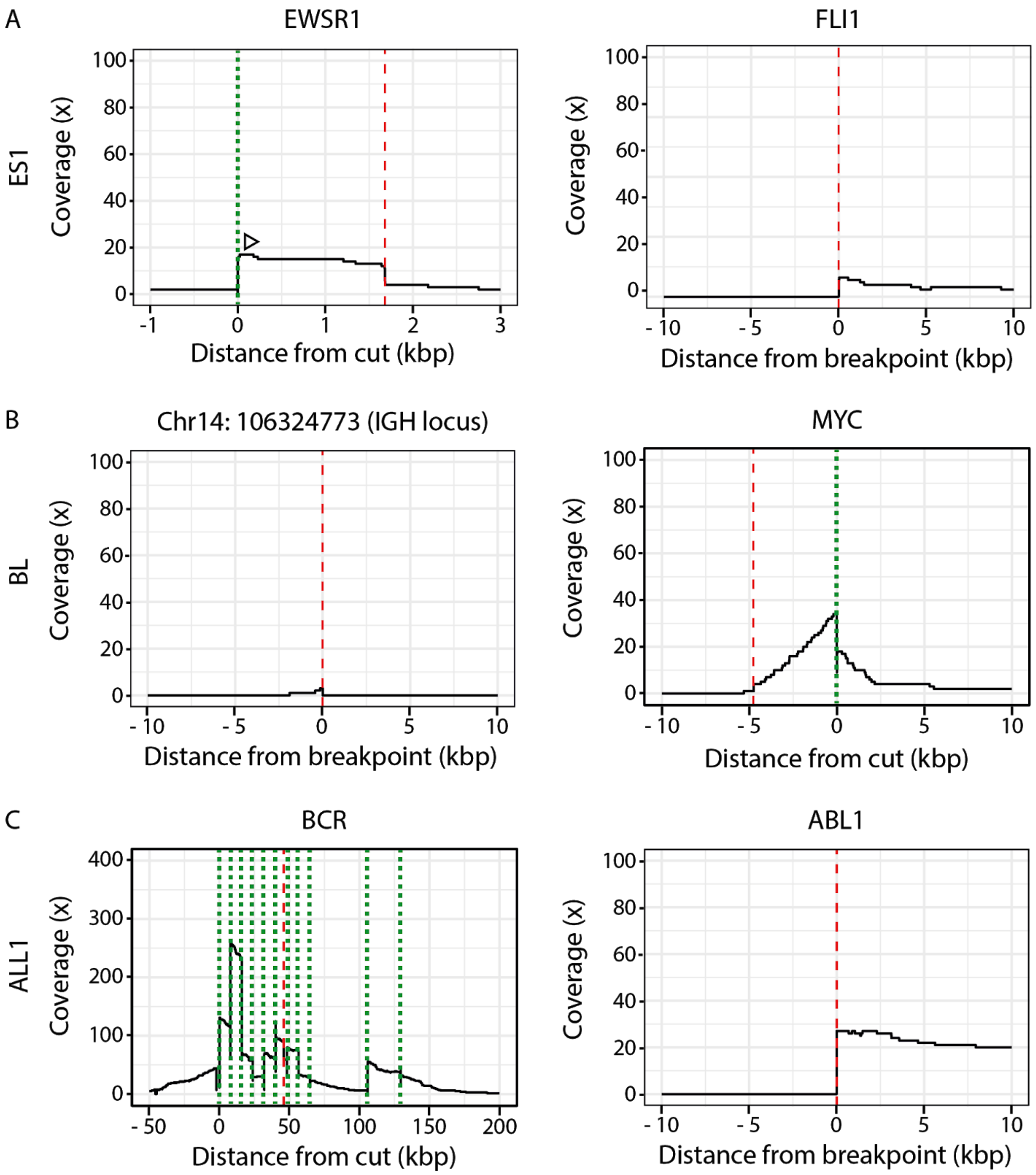

Supplementary Figure 4:

Coverage plots for the (**A**) ES1 tumor sample for the two fusion partners *EWSR1* (targeted) and *FLI1*, (**B**) BL tumor sample the *MYC* gene (targeted) and IGH locus, and (**C**) for the ALL1 sample for the two fusion partners *BCR* (targeted) and *ABL1*. Dotted lines (green) indicate cut position, dashed lines (red) indicate breakpoint positions and arrows indicate the desired read direction.

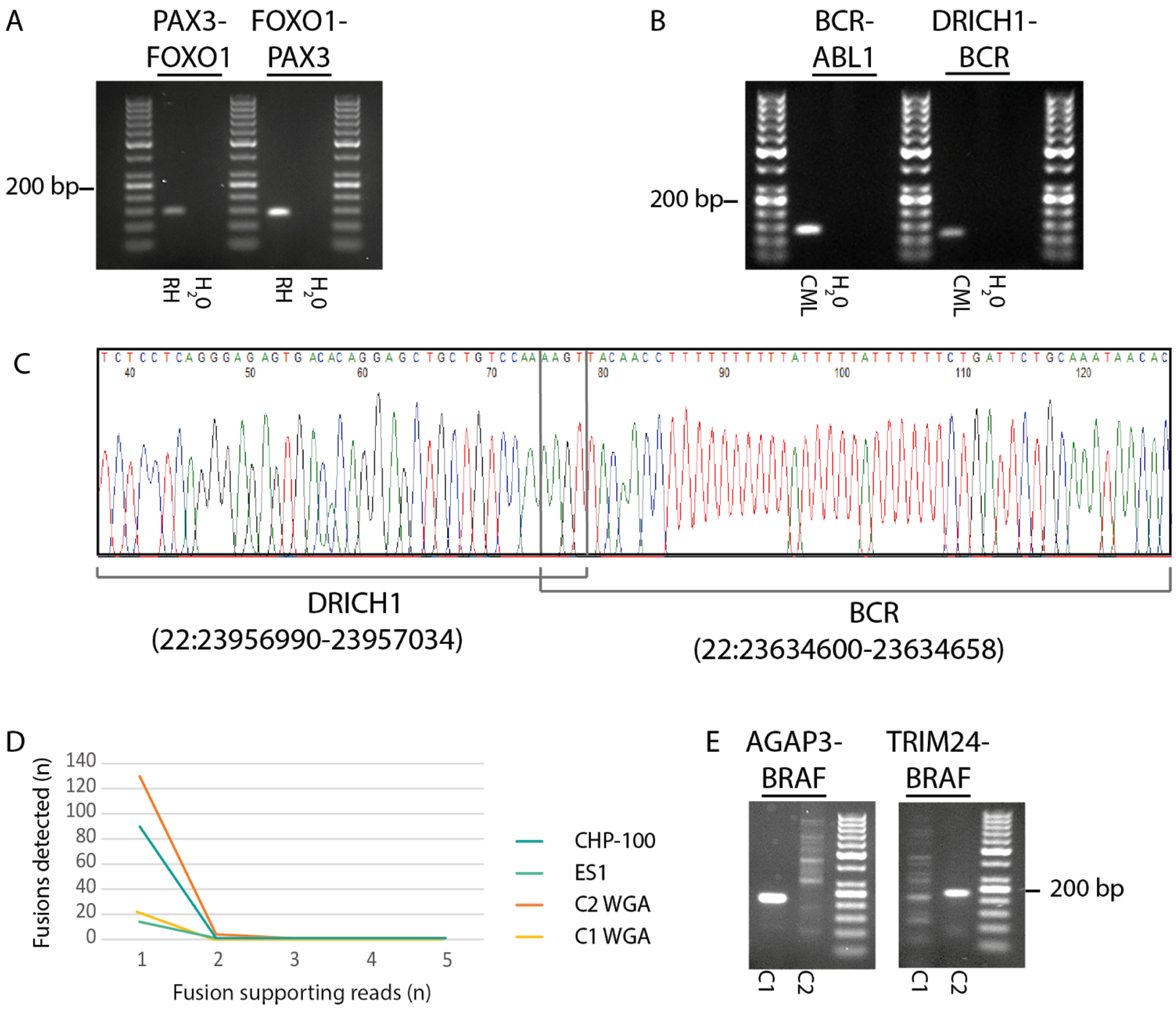

<u>Supplementary Figure 5:</u>

(**A**) Breakpoint PCR (n=1) for the reciprocal *PAX3-FOXO1* and *FOXO1-PAX3* fusion genes in the RH tumor sample. (**B**) Breakpoint PCR (n=1) for the *BCR-ABL1* and *DRICH1-BCR* fusions in the CML tumor sample. (**C**) Sanger sequencing trace for the *DRICH1-BCR* fusion gene identified in the CML sample. (**D**) Graph depicting the number of fusion genes called by NanoFG depending on the set threshold of fusion-supporting reads (n=1-5) for the samples CHP-100, ES1, C1-WGA and C2-WGA. (**E**) Breakpoint PCR (n=1) for the *AGAP3-BRAF* and *TRIM24-BRAF* fusion genes in the non-amplified tumor material of C1 and C2.

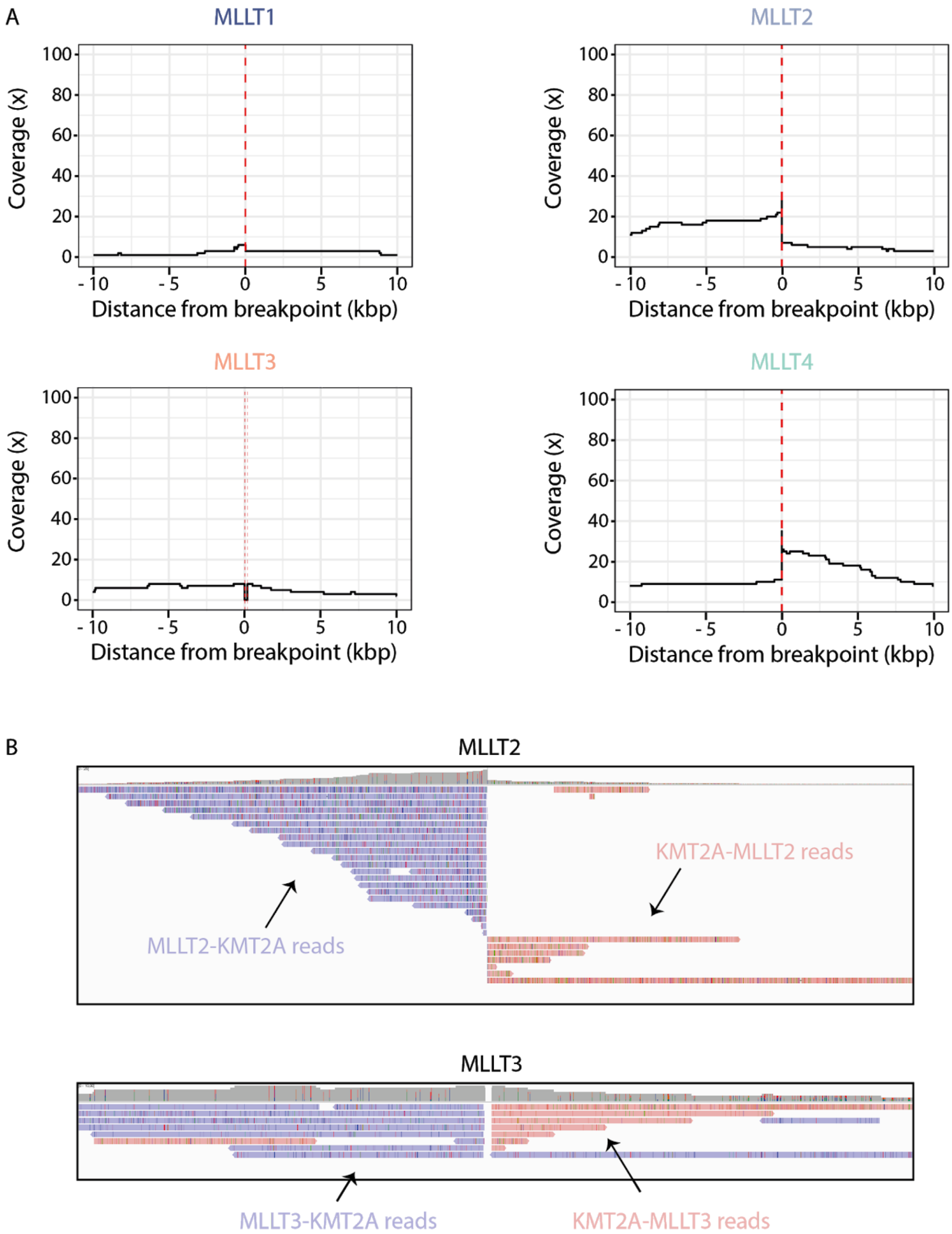

Supplementary Figure 6:

(**A**) Coverage plots for the *KMT2A* fusion partners *MLLT1*, *MLLT2*, *MLLT3* and *MLLT4*. Dashed lines (red) indicate breakpoint positions. (**B**) IGV screenshot showing reads identifying the *KMT2A-MLLT2* and *KMT2A-MLLT3* fusion genes (red) and their reciprocal translocations (blue).

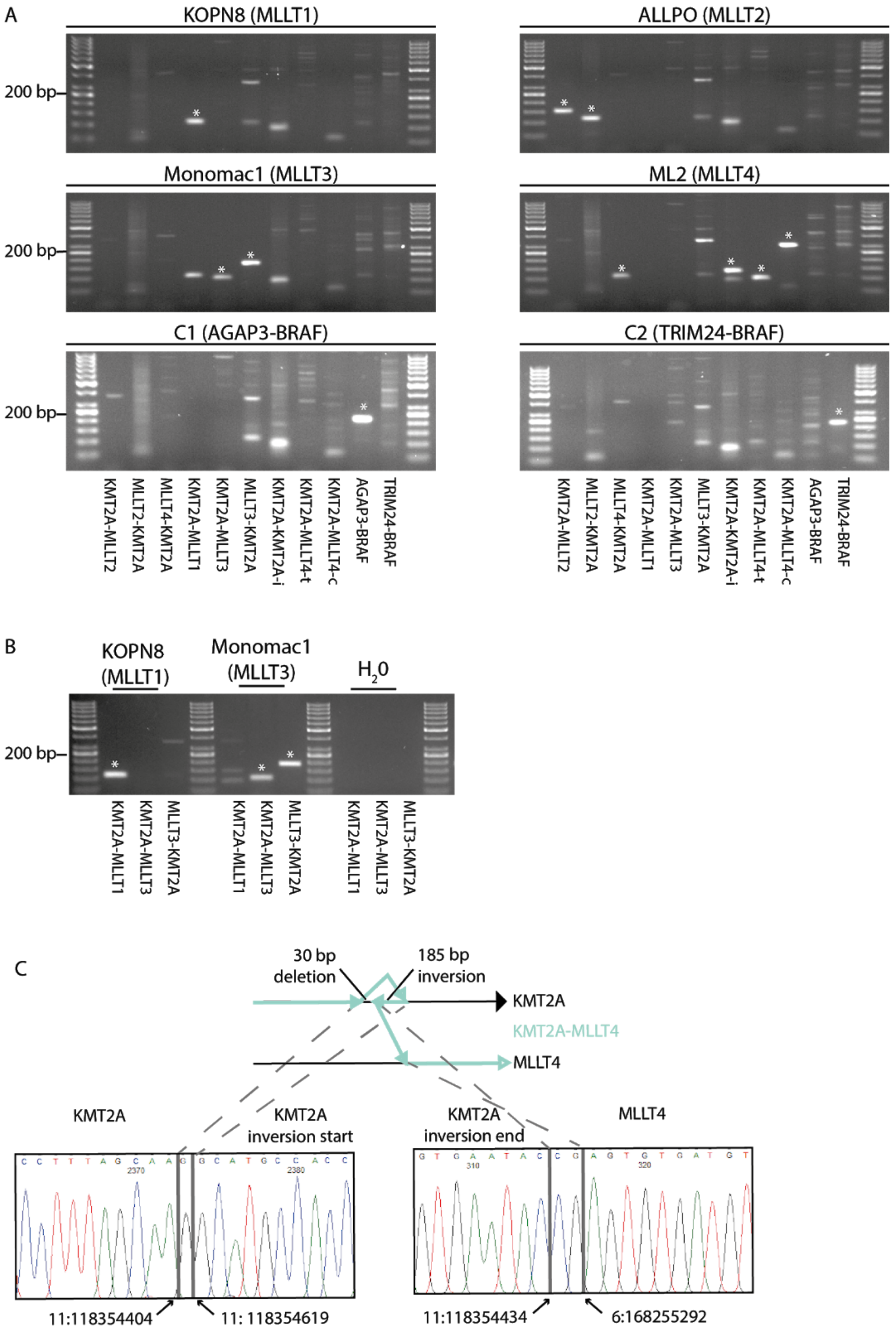
A
KOPN8 (MLLT1)
ALLPO (MLLT2)
Monomac1 (MLLT3)
ML2 (MLLT4)
C1 (AGAP3-BRAF)
C2 (TRIM24-BRAF)
200 bp
KMT2A-MLLT2
MLLT2-KMT2A
MLLT4-KMT2A
KMT2A-MLLT1
KMT2A-MLLT3
MLLT3-KMT2A
KMT2A-KMT2A-i
KMT2A-MLLT4-t
KMT2A-MLLT4-c
AGAP3-BRAF
TRIM24-BRAF
B
KOPN8 (MLLT1)
Monomac1 (MLLT3)
H2O
200 bp
KMT2A-MLLT1
KMT2A-MLLT3
MLLT3-KMT2A
C
30 bp deletion
185 bp inversion
KMT2A
KMT2A-MLLT4
MLLT4
KMT2A
KMT2A inversion start
KMT2A inversion end
MLLT4
11:118354404
11: 118354619
11:118354434
6:168255292

Supplementary Figure 7:

(**A**) Breakpoint PCR (n=1) with the fusion-specific breakpoint primers for the cell lines KOPN8 (*MLLT1*), ALLPO (*MLLT2*), Monomac-1 (*MLLT3*), ML2 (*MLLT4*) and the tumor samples C1 (*AGAP3-BRAF*) and C2 (*TRIM24-BRAF*). * indicates the bands at the correct height in the correct sample. For the complex *KMT2A-MLLT4* fusion, primers that span the inversion (KMT2A-MLLT4-i), translocation (KMT2A-MLLT4-t) and the complete rearrangement (KMT2A-MLLT4-c) were tested. (**B**) Breakpoint PCR (n=1) for the KOPN8 and freshly isolated Monomac-1 cell lines for the MLLT1 and MLLT3 fusion genes. Asterisk indicates the bands at the correct height in the correct sample. (**C**) Schematic of rearranged *KMT2A-MLLT4* fusion gene. Sanger-traces showing the exact breakpoints (vertical lines) and breakpoint-positions. The discordant nucleotides between breakpoints ("G" between *KMT2A/KMT2A* inversion; "CG" between *KMT2A* inversion/*MLLT4*) were most likely introduced during non-homologous end joining.

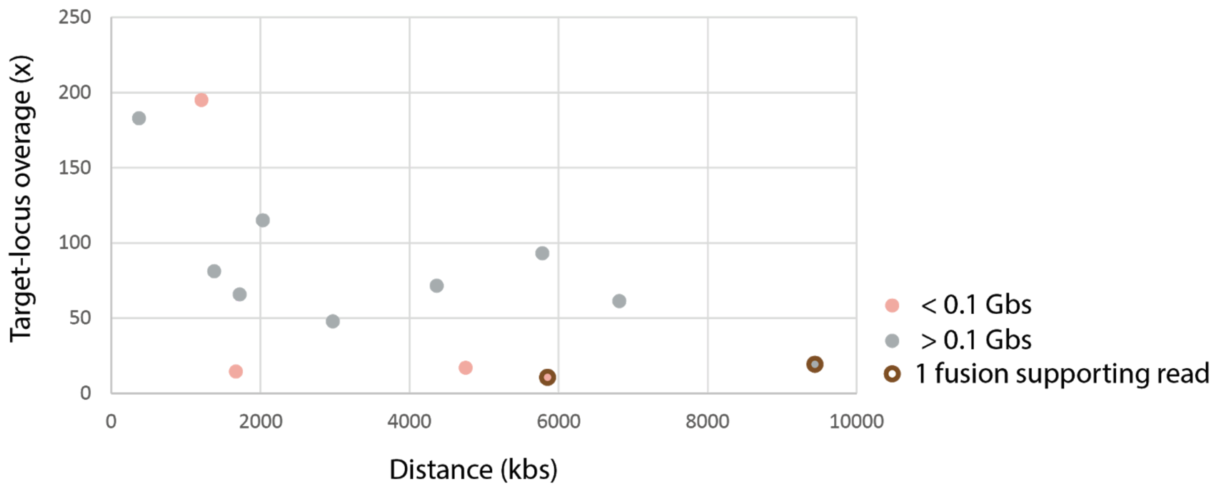

Supplementary Figure 8:

Plot depicting the relationship between coverage at the target-locus, distance between the cut and the breakpoint, and sequencing throughput. Samples are highlighted according to <0.1 Gbs (red) or >0.1 Gbs (grey) throughput. Samples with one fusion supporting read are circled in brown.
