# Supplementary material for: Partner independent fusion gene detection by multiplexed CRISPR-Cas9 enrichment and long read nanopore sequencing
Source: Nat Commun. 2020 Jun 5;11:2861. doi: 10.1038/s41467-020-16641-7 (PMC7275081; doi:10.1038/s41467-020-16641-7)
Supplement: Supplementary file 4 — Reporting Summary [file 41467_2020_16641_MOESM4_ESM.pdf]

nature research

Corresponding author(s): Glen Monroe

Last updated by author(s): 4 May 2020

Reporting Summary

Nature Research wishes to improve the reproducibility of the work that we publish. This form provides structure for consistency and transparency in reporting. For further information on Nature Research policies, seeAuthors & Referees and theEditorial Policy Checklist .

Statistics

For all statistical analyses, confirm that the following items are present in the figure legend, table legend, main text, or Methods section.

n/a	Confirmed	
☐	☒	The exact sample size (*n*) for each experimental group/condition, given as a discrete number and unit of measurement
☒	☐	A statement on whether measurements were taken from distinct samples or whether the same sample was measured repeatedly
☒	☐	The statistical test(s) used AND whether they are one- or two-sided<br>*Only common tests should be described solely by name; describe more complex techniques in the Methods section.*
☒	☐	A description of all covariates tested
☒	☐	A description of any assumptions or corrections, such as tests of normality and adjustment for multiple comparisons
☒	☐	A full description of the statistical parameters including central tendency (e.g. means) or other basic estimates (e.g. regression coefficient) AND variation (e.g. standard deviation) or associated estimates of uncertainty (e.g. confidence intervals)
☒	☐	For null hypothesis testing, the test statistic (e.g. *F*, *t*, *r*) with confidence intervals, effect sizes, degrees of freedom and *P* value noted<br>*Give P values as exact values whenever suitable.*
☒	☐	For Bayesian analysis, information on the choice of priors and Markov chain Monte Carlo settings
☒	☐	For hierarchical and complex designs, identification of the appropriate level for tests and full reporting of outcomes
☒	☐	Estimates of effect sizes (e.g. Cohen's *d*, Pearson's *r*), indicating how they were calculated

Our web collection on statistics for biologists contains articles on many of the points above.

Software and code

Policy information about availability of computer code

Data collection	Following targeted enrichment described in this manuscript, nanopore sequencing data was collected via ONT standard protocols. The library from one tumour sample was loaded onto one Flow Cell (R 9.4, ONT) according to the manufacturer's protocol. Sequencing was performed on a GridION X5 instrument (ONT) and basecalling was performed by Guppy (ONT).
Data analysis	Detection of gene fusions was performed by the bioinformatics pipeline NanoFG, described in this manuscript. NanoFG requirements, readme, and pipeline are at https://github.com/SdeBlank/NanoFG.<br><br>Reads were mapped to the human reference genome version GRCHh37 by using minimap2 (v. 2.6) and the produced SAM file was compressed to bam format and indexed with samtools (v. 1.7). Next, structural variations were detected from the bam file with either NanoSV (v. 1.2.4) or Sniffles(v.1.0.9). NanoFG selected candidate SVs that possibly form a fusion gene by annotating both ends of an SV with genes from the ENSEMBL database. If both ends of the SV are positioned in different genes it was flagged as a possible fusion. Next, all the reads supporting the candidate SVs were extracted with samtools (v. 1.7). All reads extracted per candidate fusion gene were re-mapped using LAST (921). Then, NanoSV was used to accurately define the breakpoints in the remapped fusion candidates.

For manuscripts utilizing custom algorithms or software that are central to the research but not yet described in published literature, software must be made available to editors/reviewers. We strongly encourage code deposition in a community repository (e.g. GitHub). See the Nature Research guidelines for submitting code & software for further information.

Data

Policy information about availability of data

All manuscripts must include a data availability statement. This statement should provide the following information, where applicable:

- Accession codes, unique identifiers, or web links for publicly available datasets
- A list of figures that have associated raw data
- A description of any restrictions on data availability

Data availability
Low coverage WGS Binary Alignment Map (BAM) files from nanopore sequencing are available through controlled access at the European Genome-phenome Archive (EGA), hosted at the EBI and the CRG (https://ega-archive.org), with accession number EGAS00001003964. Requests for data access will be evaluated by the UMCU Department of Genetics Data Access Board (EGAC00001000432) and transferred on completion of a material transfer agreement and authorization by the medical ethical committee of the UMCU to ensure compliance with the Dutch medical research involving human subjects act.

The ENSEMBL database for genome build GRCh37 can be found at https://grch37.ensembl.org/index.html.

Field-specific reporting

Please select the one below that is the best fit for your research. If you are not sure, read the appropriate sections before making your selection.

☒ Life sciences ☐ Behavioural & social sciences ☐ Ecological, evolutionary & environmental sciences

For a reference copy of the document with all sections, see nature.com/documents/nr-reporting-summary-flat.pdf

Life sciences study design

All studies must disclose on these points even when the disclosure is negative.

Sample size	This study was a proof-of-principle establishing the feasibility of using FUDGE for fusion gene detection. Numerous cancer types with varying fusion genes and breakpoints were used, depending on availability of DNA from collaborators. No sample size calculation was performed, however, to ensure that the technical and bioinformatic pipeline of FUDGE was not crRNA or gene specific, we included a variety of cancer types, fusion genes, and unique breakpoints. FUDGE performed well on these targeted ten recurrent fusion partners within eight solid and hematological tumor specimens and identified 22 unique fusion gene configurations.
Data exclusions	No data was excluded from this analysis.
Replication	Detection of fusion genes was the primary aim of this study and not validation of the reproducibility of the assay. However, for fusions that were detected by FUDGE with a low amount of fusion-spanning reads, we performed a breakpoint PCR with breakpoint specific primers to validate the presence of the fusion.
Randomization	Randomization was not relevant for this study as it was a proof-of-principle of the method of FUDGE to detect fusion genes.
Blinding	Assessment of two samples was done in a blinded manner as described in the text to evaluate if FUDGE could detect unknown fusions. In K1, the gene fusion was not previously detected by diagnostic efforts. In K2, the sample to be sequenced was randomly chosen by the technician and blinded from those doing downstream analysis using NanoFG for fusion gene detection.

Reporting for specific materials, systems and methods

We require information from authors about some types of materials, experimental systems and methods used in many studies. Here, indicate whether each material, system or method listed is relevant to your study. If you are not sure if a list item applies to your research, read the appropriate section before selecting a response.

Materials & experimental systems

n/a	Involved in the study	
☒	☐	Antibodies
☐	☒	Eukaryotic cell lines
☒	☐	Palaeontology
☒	☐	Animals and other organisms
☐	☒	Human research participants
☒	☐	Clinical data

Methods

n/a	Involved in the study	
☒	☐	ChIP-seq
☒	☐	Flow cytometry
☒	☐	MRI-based neuroimaging

Eukaryotic cell lines

Policy information about cell lines

Cell line source(s)	Is indicated in the manuscript. The colon cancer samples were kindly provided by Prof Ijzermans, Dept of Surgery, Erasmus Medical Center Rotterdam, The Netherlands. The Ewing sarcoma cell lines A4573(14) and CHP-100(15) harbor the EWSR1-FLI1 fusion gene and the synovial sarcoma HS-SYII cell line contains a SS18-SSX1 fusion(16). Origin of each cell line is indicated in the referenced publication.
Authentication	Authentication of each cell line was performed by using FUDGE to detect the fusion gene that the cell line was known to harbor.
Mycoplasma contamination	All cell lines tested negative for mycoplasma contamination
Commonly misidentified lines (See ICLAC register)	No commonly misidentified lines were used in this study.

Human research participants

Policy information about studies involving human research participants

Population characteristics	The study population consists of a healthy donor and patients with assorted types of cancer and various previously identified gene fusions. Other parameters were not valid for this study, only the presence of a gene fusion. Patients with different fusions and/or breakpoints were specifically sought after, to evaluate the effectiveness of different crRNAs at diverse genomic loci and highlight the breadth of this technique. Notably, none of the sequenced DNA samples used in these experiments was specifically obtained at one center or isolated for long-read sequencing. No evident self-selection bias at the population level, genomic loci, or DNA isolation technique is apparent.
Recruitment	Patients had previously been admitted to the diagnostic trajectories of several hospitals.
Ethics oversight	The healthy donor (PP) provided written informed consent. The patients ES1 and RH had been registered and treated according to German trial protocols of the German Society of Pediatric Oncology and Hematology (GPOH). This study was conducted in accordance with the Declaration of Helsinki and Good Clinical Practice, and informed consent was obtained from all patients or their guardians. Collection and use of patient specimen was approved by the institutional review boards of Charité Universitätsmedizin Berlin. Specimen, clinical data were archived and made available by Charité-Universitätsmedizin Berlin.<br>C1 and C2 were previously sequenced 21 and were kindly provided by Prof Ijzermans, Dept of Surgery, Erasmus Medical Center Rotterdam, The Netherlands.<br>B1 was a kind gift from Prof. dr. C.M. Zwaan, Erasmus Medical Center – Sophia Children's Hospital, Rotterdam, The Netherlands / Princess Maxima Center for Pediatric Oncology, Utrecht, The Netherlands. Informed consent is given by the patient or his/her parents or legal guardians, and all is performed in line with the declaration of Helsinki, and the Erasmus MC – Sophia Children's Hospital approved the experiments.<br>CML, BL, ALL1, ALL2, and B2 were from the diagnostic sample archive of the Princess Máxima Center for Pediatric Oncology, Utrecht, The Netherlands. As the work was interpreted as falling within the scope of diagnostic service improvement, it did not require specific research ethics committee approval as stated in the EU Clinical Trials Directive (2001/20/EC).

Note that full information on the approval of the study protocol must also be provided in the manuscript.
