# Supplementary material for: Partner independent fusion gene detection by multiplexed CRISPR-Cas9 enrichment and long read nanopore sequencing
Source: Nat Commun. 2020 Jun 5;11:2861. doi: 10.1038/s41467-020-16641-7 (PMC7275081; doi:10.1038/s41467-020-16641-7)
Supplement: Supplementary file 5 — Description of Additional Supplementary Files [file 41467_2020_16641_MOESM5_ESM.pdf]

Title: Supplementary Data 1
Description: Sequencing information for each sample included in this study, including coverage and enrichment values and breakpoint location and sequence.
